# A Vaccine against Nicotine for Smoking Cessation: A Randomized Controlled Trial

**DOI:** 10.1371/journal.pone.0002547

**Published:** 2008-06-25

**Authors:** Jacques Cornuz, Susanne Zwahlen, Walter Felix Jungi, Joseph Osterwalder, Karl Klingler, Guy van Melle, Yolande Bangala, Idris Guessous, Philipp Müller, Jörg Willers, Patrik Maurer, Martin F. Bachmann, Thomas Cerny

**Affiliations:** 1 Department of Internal Medicine, Lausanne University Hospital, Lausanne, Switzerland; 2 Department of Ambulatory Care and Community Medicine, Lausanne University Hospital, Lausanne, Switzerland; 3 Department of Internal Medicine, Kantonsspital, St.Gallen, Switzerland; 4 Emergency Department, Kantonsspital, St.Gallen, Switzerland; 5 Lung Center Hirslanden, Zürich, Switzerland; 6 Institute of Social and Preventive Medicine, University of Lausanne, Lausanne, Switzerland; 7 Cytos Biotechnology AG, Schlieren, Switzerland; Dalhousie University, Canada

## Abstract

**Background:**

Tobacco dependence is the leading cause of preventable death and disabilities worldwide and nicotine is the main substance responsible for the addiction to tobacco. A vaccine against nicotine was tested in a 6-month randomized, double blind phase II smoking cessation study in 341 smokers with a subsequent 6-month follow-up period.

**Methodology/Principal Findings:**

229 subjects were randomized to receive five intramuscular injections of the nicotine vaccine and 112 to receive placebo at monthly intervals. All subjects received individual behavioral smoking cessation counseling. The vaccine was safe, generally well tolerated and highly immunogenic, inducing a 100% antibody responder rate after the first injection. Point prevalence of abstinence at month 2 showed a statistically significant difference between subjects treated with Nicotine-Qβ (47.2%) and placebo (35.1%) (*P* = 0.036), but continuous abstinence between months 2 and 6 was not significantly different. However, in subgroup analysis of the per-protocol population, the third of subjects with highest antibody levels showed higher continuous abstinence from month 2 until month 6 (56.6%) than placebo treated participants (31.3%) (OR 2.9; *P* = 0.004) while medium and low antibody levels did not increase abstinence rates. After 12 month, the difference in continuous abstinence rate between subjects on placebo and those with high antibody response was maintained (difference 20.2%, *P* = 0.012).

**Conclusions:**

Whereas Nicotine-Qβ did not significantly increase continuous abstinence rates in the intention-to-treat population, subgroup analyses of the per-protocol population suggest that such a vaccination against nicotine can significantly increase continuous abstinence rates in smokers when sufficiently high antibody levels are achieved. Immunotherapy might open a new avenue to the treatment of nicotine addiction.

**Trial Registration:**

Swiss Medical Registry 2003DR2327; ClinicalTrials.gov NCT00369616

## Introduction

Despite the known health risks, people continue to smoke and use tobacco primarily as a result of their addiction to nicotine [1]. Most smokers trying to quit on their own fail on the long term [2]. Different types of counseling and behavioral therapies can increase abstinence rates [2]. Similarly, pharmacotherapies prescribed in smoking cessation interventions, such as nicotine replacement products, bupropion and the recently approved varenicline, help smokers quit, but all current therapies have only modest efficacy [2]. Consequently, there is a need for alternative and improved treatments [3].

One novel approach is provided by immunization against nicotine. The rationale is to induce antibodies which bind nicotine in the blood, thereby preventing it from crossing the blood-brain barrier [4], [5]. Thus, the reinforcing action of nicotine in the brain, which is the driving force in nicotine addiction and tobacco smoking, should be reduced. Nicotine is a small non-immunogenic molecule and must be conjugated to a carrier protein to induce antibodies. Such nicotine conjugates have been shown to induce enough antibodies in animals to sequester the drug in the blood [4], [6] attenuate nicotine addiction [7] and prevent reinstatement of nicotine seeking behavior in vaccinated animals [8].

A candidate vaccine against nicotine has been developed based on a virus-like particle (VLP)-nicotine conjugate [9]. The presentation of an antigen in a highly ordered, repetitive array, such as protein shells or coats of certain viruses, provokes strong antibody responses [10]. The coat protein of the bacteriophage Qβ forms non-infectious VLPs when expressed recombinantly in *Escherichia coli* [11]. Using chemical cross-linkers, any antigen can be placed directionally onto the VLP surface, rendering it highly immunogenic. Antigens coupled to such VLPs induce potent and long-lived antibody responses in mice [12] as well as humans [9], [13], [14]. Specific antibodies of the IgG but not IgE isotype can be detected, demonstrating that potent antibody responses may be induced in the absence of isotypes causing allergic problems. For the present vaccine, a nicotine derivative was chemically linked to VLPs formed by the coat protein of the bacteriophage Qβ. In pre-clinical animal studies, this Nicotine-Qβ vaccine induced strong and specific IgG antibody responses [9].

In a phase I study 32 healthy non-smokers were immunized with the Nicotine-Qβ vaccine at doses of 50 µg and 100 µg in presence or absence of Alum, one of the adjuvants approved for use in humans [9]. A single injection induced an anti-nicotine response in 100% of subjects, antibody levels were boosted by either a second injection or by the addition of Alum, and the vaccine was well tolerated. Based on these encouraging results, we performed an exploratory phase II randomized trial in smokers ready to quit.

We present the results of the 6-month randomized double blind trial and the subsequent 6-month follow-up period of this trial assessing immunogenicity, efficacy, safety and tolerability of the vaccine against nicotine.

## Methods

### Design overview

The protocol synopsis for this trial and supporting CONSORT checklist are available as supporting information; see Checklist S1 and Protocol S1.

We performed a phase II randomized, double blind, placebo-controlled trial. The study subjects were recruited through public advertisements on billboards posted in the three clinical study centers in Switzerland (Kantonsspital, St. Gallen, University Hospital Center, Lausanne, and Hirslanden Lung Center, Zurich) where the study was performed. Interested participants were asked to call the study center. The trial was explained and a pre-screening interview was undertaken. If the subjects met the initial screening criteria, they were scheduled for a visit to provide informed consent and to undergo a screening of their health and a medical examination including standard clinical laboratory and electrocardiogram. Participants did not receive any compensation.

We considered three main objectives. First, to assess the clinical efficacy of Nicotine-Qβ in smokers willing to quit, second, to evaluate the safety and tolerability of Nicotine-Qβ in smokers and third, to determine the immunogenicity of Nicotine-Qβ.

### Participants

Participants were required to be between 18 and 70 years, to have been smoking at least 10 but no more than 40 cigarettes/day for more than 3 years, have a Fagerström Score of at least 5 at screening [15], and willing to quit smoking. Women of childbearing potential had to agree to use an effective form of contraception during treatment and up to 12 months after the last dose of the vaccine. Exclusion criteria were the following: cardiovascular, renal, pulmonary, endocrine, or neurological disorders, ulcers, skin disorders, autoimmune diseases or severe allergies; risk behavior to acquire HIV; an active liver infectious disease; a current diagnosis or a history of major depressive episodes, of panic attacks, psychosis, bipolar or eating disorders; use of other smoking-cessation treatments, like bupropion or nicotine replacement therapy within 6 months before study enrollment or at the time of screening; pregnancy or lactation; abuse of alcohol or other recreational drugs; use of a psychoactive drug (excluding sleeping pills) within one month before enrollment; and regular use of any non-cigarette tobacco product.

We obtained written informed consent from all subjects before they were enrolled in the study. The Ethics Committees of the three centers (i.e., Lausanne Medical School Ethics Committee, St Gallen: Ethics Committee of the Canton St. Gallen, Hirslanden: Ethics Committee of the Canton Zurich) approved the study. The Swiss health authorities were notified of its conduct and the study protocol has been registered in the Swiss Medical Registry (Swissmedic # 2003DR2327) and at www.clinicaltrials.org (NCT 00369616).

### Randomization and Interventions

Two thirds of the subjects were scheduled to receive five intra-muscular injections of 100 µg Nicotine-Qβ in Alum and one third to receive indistinguishable placebo (Alum alone) at monthly intervals, i.e., on months 0, 1, 2, 3, and 4. After having fulfilled the eligibility criteria, investigators sent the subject's identification number to the local pharmacist, who assigned subjects to treatment according to a randomization list, prepared using standard software, with a block size of 15. All study personnel, participants, study statisticians and data monitoring committee were blinded to treatment assignment for the duration of the study. A “target quitting date” was set at 1 month after the first vaccination. Individual standardized counseling was provided weekly to all study participants starting at week 3 after the first vaccination until week 16 by health care professionals and physicians trained through an effective smoking cessation program [16]. The initial phase ended at month 6 and was followed by an additional 6-month period of follow-up with two visits at months 9 and 12. Neither additional injections nor counseling was given during the follow-up phase.

The active pharmaceutical agent was Nicotine-Qβ, i.e., a nicotine derivative coupled to the VLP Qβ as a carrier. The VLP is produced by recombinant expression of the coat protein of the bacteriophage Qβ in *E. coli*. The adjuvant used with Nicotine-Qβ was Alum (Alhydrogel: Brenntag Biosector A/S, Frederikssund, Denmark), and the placebo consisted of Alhydrogel alone. All materials for the clinical trial were produced to current good manufacturing principles according to the International Conference on Harmonization guidelines. An injection volume of 2 ml was given intramuscularly at the upper arm. The vaccine dose was selected based on the results from the Phase I study which showed an early onset, a maximal immune response with 100 µg Nicotine-Qβ and an about twofold increase of titers by the addition of Alum. Additional information on the product, pre-clinical toxicological safety studies, animal efficacy studies and the ELISA are published elsewhere [9].

### Outcomes and follow-up

The first primary outcome was abstinence from smoking defined as self-reported abstinence from smoking, confirmed by a carbon monoxide concentration in expired air of less than 10 ppm. Carbon monoxide was measured pre-study, at each monthly visit after vaccination and during follow-up at months 9 and 12 with a Micro Smokerlyzer® (Bedfont). Study participants were considered to be *continuously abstinent* when at all monthly visits from month 3 until month 6 they declared themselves as being non-smokers during that period and when the carbon monoxide concentration in their exhaled air was below 10 ppm. We did not use cotinine as a second confirmation means of abstinence because the binding of nicotine to antibodies might prolong the elimination of nicotine and its metabolite cotinine [17]. *Point prevalence* of smoking or of abstinence was defined at each assessment visit as the smoking status (self-report confirmed by CO below 10 ppm) of a study participant at that visit, irrespective of his/her smoking status on the visits before or after. Self-reported cigarette consumption was recorded daily in paper and pencil diaries. Subjects with missing visits or who were lost to follow-up at any time during the study were considered as smokers. Given the exploratory nature of this Phase II trial, the study protocol stipulated that additional evaluations guided by the results might be considered, in particular the correlation between the clinical outcome and anti-nicotine antibody titers could be assessed.

The other primary outcomes were immunogenicity, safety and tolerability of Nicotine-Qβ. The immunogenicity was assessed by determination of specific anti-nicotine antibodies of IgG isotype by ELISA using an RNAse-nicotine conjugate [9] in sera before vaccination and then at monthly intervals up to month 6 and at months 9 and 12. Since a human anti-nicotine monoclonal IgG reference standard was not available, nicotine-specific IgG levels are reported as titers. The ELISA titer for each serum specimen corresponds to the dilution needed to achieve an optical density of 50% of the optical density reached at saturation. An antibody responder was defined as a subject who had an anti-nicotine IgG titer, which was larger than the unspecific background reactivity (average plus three standard deviations) of the ELISA. Background reactivity of the ELISA was determined using preimmune sera. Sub-group analyses were performed by using the area under the curve (AUC) of log-transformed titers from month 3 to month 6.

Safety and tolerability were assessed through systematic collection of vital signs and all reported symptoms, as well as standard clinical laboratory and injection site examination in all subjects. A specific safety check-up was performed one week after each injection. Subjects also kept a diary for self-assessment of local reactions. Information was collected about adverse events that occurred during the double blind and follow-up periods. An adverse event was any new undesirable medical occurrence, which did not necessarily have to have a causal relationship with this treatment. The classification of the severity was based on the following scale. *Mild*: the adverse event was noticeable to the individual; it did not require modification of the dose but may have required additional therapy, such as paracetamol. *Moderate*: the adverse event interfered with the individual's daily activities; it may have required additional therapy, but did not require discontinuation of the study agent. *Severe*: the adverse event was intolerable and necessitated additional therapy or discontinuing the study agent. A *serious adverse event* was any untoward medical occurrence that at any dose resulted in death, in persisting or significant disability/incapacity, was life-threatening, required in-patient hospitalization, or any significant medical event as judged by the investigators.

To assess craving and withdrawals symptoms, we used two questionnaires, the Questionnaire on Smoking Urges [18] addressing two conditions (i.e., intention to smoke and anticipation of relief from the urgent desire to smoke) and the Wisconsin Withdrawal Scale [19] addressing craving, concentration, sleeplessness, anger, anxiety, sadness and hunger.

### Statistical analyses

Statistical analyses were performed with SAS for Microsoft Windows® 9.1.3. Pearson Chi-Square test without correction was used to calculate the statistical significance of the effect of vaccination (active versus placebo) and the effect of antibody titer levels (high, medium, low versus placebo) on continuous abstinence and on point prevalence abstinence. For continuous variables differences between two groups were analyzed with the two-sample t-test or with the Mann-Whitney test as nonparametric test, and, for more than two groups, with analysis of variance or the Kruskal-Wallis test as nonparametric test. The influence of baseline parameters on the abstinence rate was tested by means of a logistic regression analysis (SAS ProcLogistic using the binary logit model). Outcomes were considered significant if p values of the respective statistical tests were smaller than 0.05. Nicotine replacement users were excluded from the per-protocol analysis.

We powered our study on rates of continuous abstinence and calculated a sample size that would detect a difference of at least 15% (smoking abstinence rates of at least 30% compared to 15% on placebo) with an alpha error of 5% or less and a power of 90% or more. We chose unbalanced group sizes with a ratio of 2∶1 for active vs. placebo treatment to allow as many study participants as possible to potentially benefit from the active treatment and to permit sub-group analyses of efficacy by antibody response. Based on theses parameters, the required sample size was 300 subjects (200 in the active group vs. 100 in the placebo group). Considering the exploratory nature of this Phase II study, additional participants were considered as valuable source of information.

## Results

### Participants

**Figure 1:**
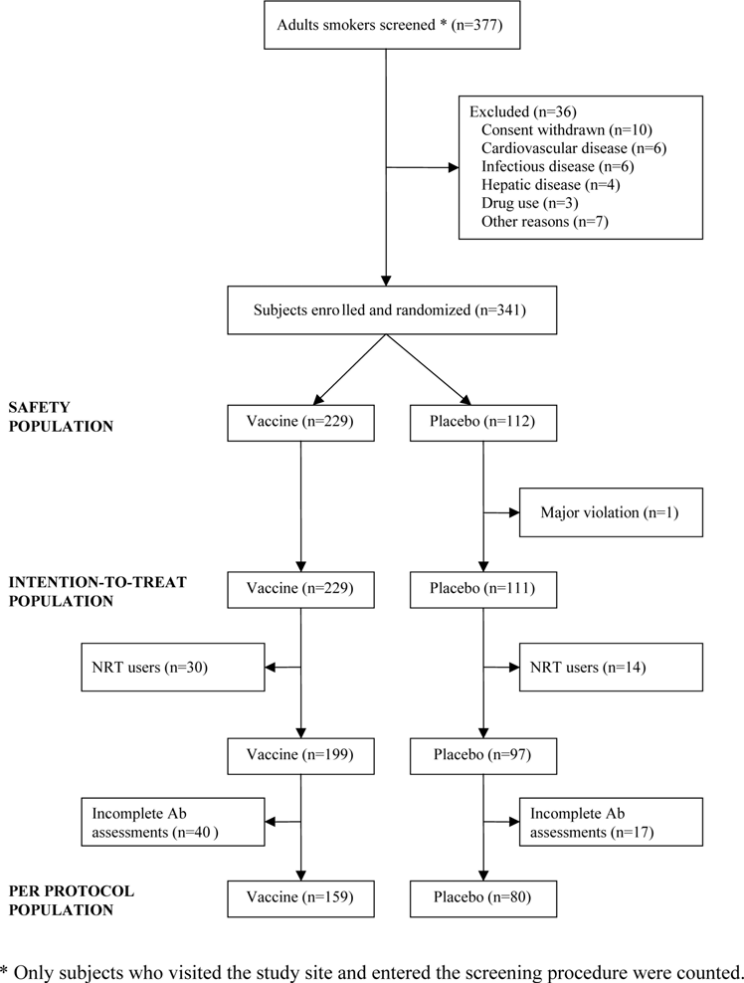
Flow Chart of Study Subjects.

Enrollment of smokers started in December 2003 and was completed in September 2004. Among the 377-screened smokers, 36 were excluded for various reasons as detailed (**Figure 1**). Altogether 341 randomized subjects received at least one dose of the study treatment (safety population). Their baseline characteristics were similar across groups (**Table 1**). One additional subject was excluded for the intention-to-treat analysis (**Figure 1**) because of a violated key inclusion criterion, namely that he had already stopped smoking at the baseline visit. Five immunizations with Nicotine-Qβ were administered at months 0, 1, 2, 3 and 4. The target quit date was set to month 1 and smoking cessation counseling started at week 3 and was performed until month 4.

**Table 1:** Baseline characteristics of subjects.

	Safety Population		Per Protocol Population*			
	*N* = 341		*N* = 239			
	Nicotine-Qβ	Placebo	Nicotine-Qβ	Nicotine-Qβ	Nicotine-Qβ	Placebo
			High	Medium	Low	
			Ab responders	Ab responders	Ab responders	
	*N* = 229	*N* = 112	*N* = 53	*N* = 53	*N* = 53	*N* = 80
Male gender, %	59	58	68	55	62	61
Age**, years	42.1 (36.6 ; 48.5)	42.1 (36.3 ; 47.9)	43.2 (36.0 ; 48.9)	41.5 (38.8 ; 46.3)	41.3 (35.6 ; 48.0)	41.5 (34.4 ; 48.2)
Number of cig. smoked/day**	25 (21 ; 32)	25 (20 ; 35)	25 (20 ; 35)	25 (22 ; 33)	25 (21 ; 30)	27 (22 ; 35)
Number of years smoked**	25 (19 ; 31)	25 (19 ; 31)	27 (19 ; 31)	24 (19 ; 31)	25 (18 ; 30)	25 (19 ; 32)
CO ppm exhaled air**	29 (20 ; 38)	27 (21 ; 40)	25 (16 ; 31)	28 (23 ; 37)	30 (20 ; 37)	28 (21 ; 40)
Fagerström score, range (1–10)**	7 (6 ; 8)	7 (6 ; 7)	7 (6 ; 8)	7 (6 ; 8)	7 (6 ; 7)	6 (6 ; 7)
Number of previous quitting attempts**	3 (2 ; 4)	3 (2 ; 4)	3 (2 ; 4)	3 (1 ; 5)	3 (2 ; 4)	3 (2 ; 4)

Ab Antibody titer.

* Nicotine replacement therapy users and subjects with missing titers at months 4 to 6 were excluded; the participants on active treatment were separated into three responder subgroups according to their antibody titers: high, medium, and low antibody titer levels.

** Median values (25% ; 75% quartiles).

### Immunogenicity

**Figure 2:**
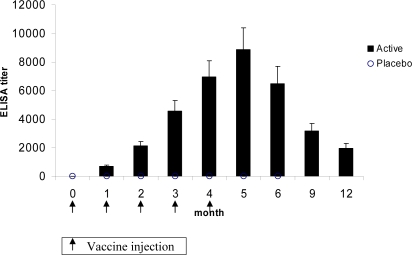
Geometric mean nicotine-specific IgG titers (±95% CI) (Active, N = 227; placebo, N = 108).

Of the 340 subjects in the intention-to-treat population, 229 received the vaccine and 111 the placebo. For 5 subjects (2 vaccine/3 placebo) only pre-immune sera were available for the immunogenicity analyses. No induction of nicotine-specific IgG antibodies was observed for subjects receiving placebo. In subjects receiving the active treatment, a 100% antibody responder rate was achieved with a single injection of Nicotine-Qβ. The second, third, fourth and fifth immunization at months 1, 2, 3 and 4 boosted the nicotine-specific IgG levels and peak titers were achieved at month 5, i.e. 4 weeks after the 5^th^ injection (**Figure 2**). Thereafter, titers declined up to month 12 with a half-life of about 90 days.

### Smoking behavior in the intention-to-treat population

Continuous abstinence rates between month 3 and month 6 were 30.1% in the vaccine group and 26.1% in the placebo group, a non-significant difference (*P* = 0.44). Point prevalence abstinence rates at month 2 were significantly different (*P* = 0.036) between vaccine group (47.2%) and placebo (35.1%). A higher point prevalence abstinence rate of subjects vaccinated was maintained up to month 6, but the differences after month 2 were not significant.

We then investigated which variables influenced the continuous abstinence rate in the intention-to-treat population by logistic regression. No significant influence of treatment, age, gender, bodyweight, number of cigarettes smoked, duration of smoking or Fagerström score was detectable (all *P*>0.1). When the anti-nicotine antibody titer at month 2 (available for 325 subjects, corresponding to 95% of the intention-to-treat population) was included in the analysis, a significant effect of the antibody titer on the continuous abstinence rate was determined (*P* = 0.027) while the other variables showed no influence. The significant influence of anti-nicotine levels on abstinence was also observed when only the subjects in the vaccine group were analyzed (*n* = 219, *P* = 0.018).

Antibodies induced by vaccination are expected to bind nicotine in the blood, reduce its passage into the brain and thus interfere with the reinforcing properties of nicotine e.g. during a lapse. Thus, vaccination is not expected to reduce craving and withdrawal during a smoking cessation attempt. Accordingly, there was no detectable difference between vaccine and placebo group on intention to smoke and on anticipation of relief from the urgent desire to smoke as addressed by the Questionnaire Smoking Urge, as well as on withdrawal symptoms using the Wisconsin Withdrawal Scale (data not shown).

### Safety and Tolerability

**Table 2** shows an overview of adverse events in the 341 subjects of the safety population. Of the 9 serious adverse events reported in 8 participants, only one might be related to the study treatment. This occurred in a 60-year old woman who reported flu-like symptoms associated with chest pain. However, there was no evidence for heart disease and at the 6-month follow-up the participant was free of any cardiovascular or pulmonary diseases. The majority of all adverse events were mild to moderate (95.7%) and only 4.3% of events were rated as severe. These were mainly concurrent infections equally distributed between active treatment and placebo groups, and systemic or local reactions, which were more prevalent on active treatment. The most prominent systemic adverse event was reported as “flu-like symptoms” by 69.4% of vaccinated subjects compared to 12.5% of placebo subjects (**Table 3**). These symptoms usually appeared 2–12 hours after injection and disappeared 24 hours post dose. There were several other adverse events which seem to be related to the flu-like syndrome but were reported separately such as pyrexia, headache, chills and myalgia with a significantly higher incidence on treatment group as specified by the OR and its 95% CI (Table 3). Severe pyrexia was observed in 3 out of 194 reports (1.5%) and flu-like symptoms were rated as severe in 7 out of 344 reports (2.0%). Paracetamol was prescribed for amelioration of symptoms if necessary. If analyzed over time, the incidence (percent of subjects affected per injection period) of flu-like symptoms showed an increase from 21% (1^st^ injection period) to 44% (2^nd^) and thereafter declined to 40% (3^rd^), 25% (4^th^) and 24% (5^th^ injection period). Among the local reactions, pain at the injection site was the most prevalent symptom, whereas local swelling, erythema and edema were rarely reported. There was no difference in the incidence of flu-like symptoms between the 3 subgroups (based on anti-nicotine antibody levels as defined below) and no significant difference in efficacy between subjects who had flu-like symptoms and those without (*P* = 0.21).

**Table 2:** Overview of Adverse Events in the safety population.

	Vaccine			Placebo			
	N = 229			N = 112			Odds ratio
	#	n	%	#	n	%	OR (95% CI)
Total AEs	1683	225	98.3	426	104	92.9	4.3 (1.3–14.7)
Mild	1149	221	96.5	279	95	84.8	4.9 (2.1–11.8)
Moderate	468	167	72.9	122	66	58.9	1.9 (1.2–3.0)
Severe	66	48	21.0	24	15	13.4	1.7 (0.9–3.2)
Serious AEs	6 *	5	2.2	3 **	3	2.7	0.8 (0.2–3.5)
Related AEs	864	199	86.9	73	41	36.6	11.5 (6.7–19.8)

# Total number of adverse events.

n Number of subjects who experienced at least one event of the respective category. A subject, who had e.g. one mild and one moderate event was counted in both categories.

(%) Percentages of subjects with at least one event, calculated on the total number of subjects in the respective groups.

OR odds ratio; CI confidence interval; AEs adverse events.

* Limb operation, Pneumonia, Head trauma, Disc prolapsed, Crime victim, Chest pain.

** Minor surgery, Osteomyelitis, depression.

**Table 3:** Adverse Events with an incidence across all 5 injections of at least 10% of subjects in either group.

	Nicotine-Qβ			Placebo			
	N = 229			N = 112			Odds ratio
	#	n	%	#	n	%	OR (95% CI)
Flu-like symptoms	325	159	69.4	19	14	12.5	15.9 (8.5–29.8)
Pyrexia	184	96	41.9	10	9	8.0	8.3 (4.0–17.1)
Headache	171	92	40.2	55	30	26.8	1.8 (1.1–3.0)
Nasopharyngitis	89	73	31.9	34	29	25.9	1.3 (0.8–2.2)
Injection site pain	68	45	19.7	3	2	1.8	13.5 (3.2–56.5)
Rigors (Chills)	47	31	13.5	0	0	0.0	—
Myalgia	46	31	13.5	6	6	5.4	2.8 (1.1–6.8)
Back pain	31	25	10.9	13	12	10.7	1.0 (0.5–2.1)
Weight increased	24	24	10.5	13	13	11.6	0.9 (0.4–1.8)
Rhinitis	26	21	9.2	19	12	10.7	0.8 (0.4–1.8)
Influenza	18	16	7.0	14	12	10.7	0.6 (0.3–1.4)

# Number of events.

n number of subjects with at least one event.

% Percentages of subjects with at least one event, calculated on the number of subjects in the respective groups.

OR odds ratio.

CI confidence interval.

### Smoking behaviour in the per protocol analysis

Given the significant effect of the antibody titer on the continuous abstinence rate, we then analyzed the per-protocol population according to antibody levels to explore the relationship between titers and efficacy. Given the highest differences in point prevalence abstinence rate between vaccine and placebo groups at month 2, we explored continuous abstinence from month 2 onwards, and used continuous abstinence from month 2 until month 6 for all exploratory evaluations. We excluded the subjects who concomitantly used nicotine replacement therapy (*n* = 44) (**Figure 1**). Nicotine replacement therapy (NRT) is a self-medication available over-the-counter in Switzerland. The study protocol stated that nicotine replacement therapy use should be discouraged since it is believed to have a different effect on the two treatment groups. NRT products, e.g. nicotine patches, release nicotine into the blood. The nicotine from NRT might saturate the nicotine-specific antibodies induced by vaccination. Accordingly, the antibodies would not be available any more for binding nicotine from a lapse and thus, the effect of the nicotine vaccine would be diminished or even abolished. Vice versa, when nicotine from the NRT is bound to antibodies, its passage to the brain is reduced and the effect of the NRT would also be diminished. In contrast, in smokers on placebo nicotine replacement therapy may exert its documented positive effect. Therefore, subjects concomitantly using NRT were excluded. After exclusion, none of the baseline characteristics were different between the groups treated with the vaccine (*n* = 199) or placebo (*n* = 97).

**Figure 3:**
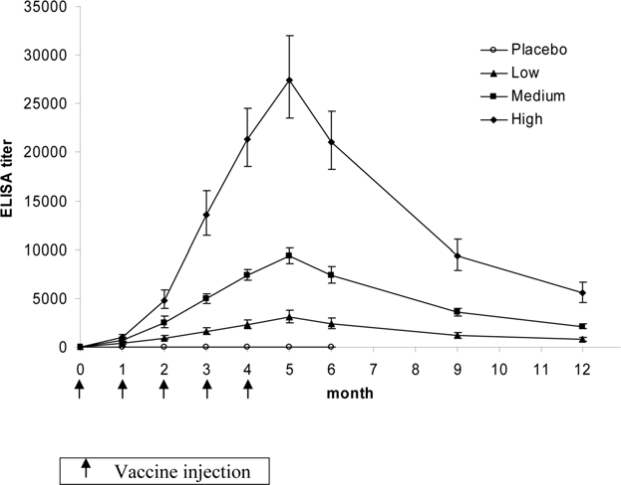
Nicotine-specific geometric mean IgG titers (±95 confidence interval) in the per protocol population (N = 229).

For the correlation with antibody titers, all subjects who had performed the scheduled visits and blood samplings were included. The individual AUC of anti-nicotine IgG titers could not be calculated for subjects with incomplete titer values because of missing one or several visits (*n* = 57). The 2∶1 randomization ratio was preserved in this per protocol population (vaccine *n* = 159, placebo *n* = 80) and baseline characteristics of excluded and included subjects were not significantly different. The 159 Nicotine-Qβ treated subjects were divided into three equal-sized categories based on AUC tertiles. Low, medium, and high responder groups were thus defined, each containing 53 subjects. We divided subjects into three equal groups to have the minimal number of points required to study the relationship between antibody titers and efficacy outcomes. The baseline characteristics of these subgroups were similar (**Table 1**) and the three groups showed a similar time course of the immune response with peak titers seen at month 5. They differed only in the magnitude of nicotine-specific IgG titers achieved (**Figure 3**).

Overall, there was a statistically significant difference in continuous abstinence rates from month 2 until month 6 among the 3 subgroups (low, medium and high titer groups) and placebo (*P* = 0.012, **Table 4**). While there was no difference between abstinence rates of subjects on placebo and the subjects with either low or medium antibody response, the difference between subjects on placebo (31.3%) and the subjects with a high antibody response (56.6%) was both clinically relevant (25.3% more abstainers in the high antibody response group) and statistically significant (*P* = 0.004). The odds ratio for continuous abstinence with high antibody titers vs. placebo was 2.9 (95% CI 1.4–5.9). Inclusion of nicotine replacement therapy users in the analysis did not change the overall outcome: there was a statistically significant overall difference (*P* = 0.025) of abstinence rates across the 3 subgroups (low-medium-high titers) and placebo. The difference between placebo (28.6%) and the participants with a high antibody response (50%) was again both clinically relevant (difference 21.4%) and statistically significant (OR 2.5 (95% CI 1.3–4.9); *P* = 0.008).

**Table 4:** Continuous abstinence rates from months 2 to 6 in the per protocol population*.

	Total	Abstainers			
	N	N	%	95% CI	P value**
Vaccinated subjects	159	64	40.3	32.6–47.9	0.174
High Ab responders	53	30	56.6	43.3–70.0	0.004
Medium Ab responders	53	17	32.1	19.5–44.6	0.920
Low Ab responders	53	17	32.1	19.5–44.6	0.920
Placebo	80	25	31.3	21.1–41.4	

CI Confidence interval.

Ab Antibody titers.

* nicotine replacement therapy users and subjects with missing titers were excluded; the participants on active treatment were separated into three responder groups according to their antibody (Ab) titers: high, medium, and low antibody titer levels.

** vs. placebo.

At 12-month follow-up, increased abstinence in the high responder group was maintained. The difference between participants on placebo (21.3%) and those with high antibody response (41.5%) was both clinically relevant (difference 20.2%) and statistically significant (OR 2.6 (95% CI 1.2–5.7); *P* = 0.012).

**Figure 4:**
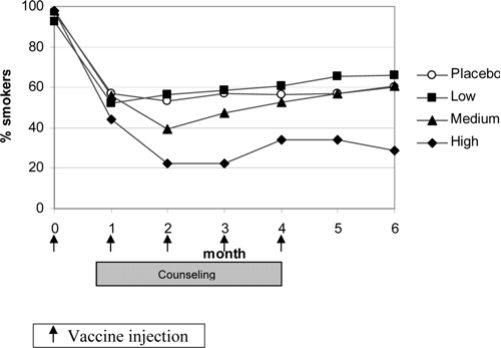
Point prevalence of smoking abstinence per antibody responder group.

Regarding point prevalence rates, the maximum effect was reached already at month 2, i.e. 4 weeks after the second dose of the vaccine (**Figure 4**). The subgroup of 53 subjects with high antibody titers showed a clear separation from placebo from month 2 onwards. At month 2, point prevalence abstinence rate was 77.4% for subjects with high antibody response and 46.8% for placebo (OR 3.9 (95% CI 1.8–8.5); *P* = 0.0005) (**Figure 4**). The difference in abstinence rates between high antibody responders and placebo was statistically significant at all time points between months 2 and 6 (all *P*<0.012). The abstinence rate of the group with medium titers separated from placebo at month 2, without reaching statistical significance (absolute difference 13.6%, *P* = 0.13), and then reverted towards placebo rates at month 6.

**Figure 5:**
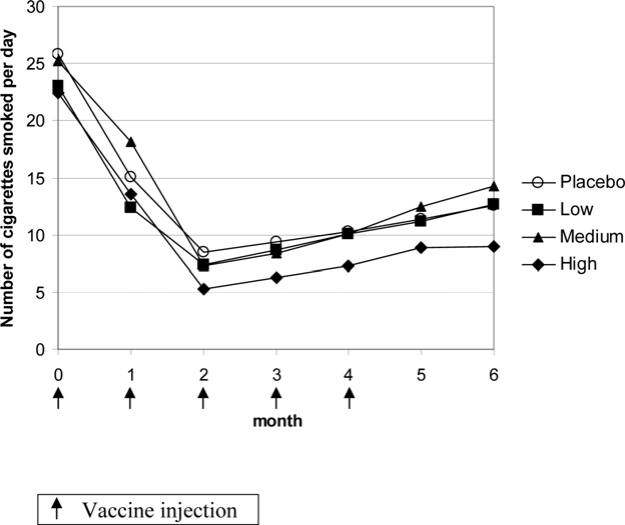
Mean daily number of cigarettes smoked by non-abstainers per antibody responder group at the respective visit.

There was no indication that some subjects might have increased their smoking with the vaccine (i.e., no compensatory smoking) (**Figure 5**). In contrast subjects who had high antibody titers, but were non-abstainers as assessed by continuous abstinence, even showed a tendency for lower cigarette consumption. Data on carbon monoxide in exhaled air were in agreement with the reduction in number of cigarettes smoked (**Table 5**).

**Table 5:** Carbon Monoxide Concentration* in Exhaled Air, per protocol population, in all subjects and per antibody responder group.

				Month					
	0	1	2	3	4	5	6	9**	12**
All subjects (N = 159), mean	28.0	5.0	3.0	3.0	3.0	4.0	6.0	12.0	12.0
Percentiles 25; 75	19; 36	2; 19	2; 15	2; 20	1; 20	1; 25	1; 23	1; 26	1; 28
								n = 153	n = 146
High (N = 53), mean	25.0	3.0	2.0	2.0	2.0	2.0	2.0	3.0	3.5
Percentiles 25; 75	16; 31	1; 14	2; 5	2; 3	1; 5	1; 6.5	1; 7	0; 17	0; 22
								n = 51	n = 50
Medium (N = 53), mean	28.0	6.0	3.0	6.0	5.0	14.0	14.0	16.0	18.5
Percentiles 25; 75	23; 37	2; 24	2; 13	2; 21	1; 21	1; 23	2; 26	2; 30	2; 27
								n = 52	n = 50
Low, (N = 53), mean	30.0	6.0	9.0	13.0	10.0	17.5	12.0	15.5	12.5
Percentiles 25; 75	20; 37	2; 23	2; 19	2; 26	2; 25	2; 29	2; 26	2; 28	2; 30
								n = 50	n = 46
Placebo, (N = 80)	28.0	7.0	5.0	8.5	12.0	14.0	14.0	18.0	19.0
Percentiles 25; 75	21; 40	2; 22	2; 19.5	2; 27.5	2; 23.5	2; 31.5	2; 30	2; 30	2; 31
								n = 77	n = 75

* Mean and percentiles 25%–75%.

** Not all subjects returned for 9- and 12-months follow-up visits.

Carbon monoxide concentration expressed in ppm.

High = Subgroup of subjects with high anti-nicotine antibody levels.

Medium = Subgroup of subjects with medium anti-nicotine antibody levels.

Low = Subgroup of subjects with low anti-nicotine antibody levels.

## Discussion

All subjects who received Nicotine-Qβ produced a nicotine-specific IgG antibody response after the first dose of Nicotine-Qβ. This response increased with further injections. No demographic, clinical or smoking-related baseline variable was found to have an effect on antibody titers.

Despite the 100% antibody responder rate, in the intention-to-treat population, vaccination against nicotine did not significantly increase continuous abstinence rates. This could have been caused by two reasons: First, sequestration of nicotine by antibodies in blood might not be sufficient for increasing abstinence rates in smokers. Second, vaccination against nicotine–similar to prophylactic vaccinations against infectious diseases-induces a certain distribution of antibody levels in the different subjects. Thus, the second reason for failure could have been that average antibodies levels induced in this study were not high enough to show a significant effect in the ITT population. The study presented here was the first clinical study testing a nicotine vaccine in a smoking cessation setting. It was therefore unknown which antibody levels had to be achieved. Two findings indicate the second reason is more likely to be correct. Logistic regression showed that antibody titer is the only variable which had a significant effect on continuous abstinence and, more importantly, subgroup analyses based on the antibody titers revealed a statistically significant and clinically relevant increase in continuous abstinence from month 2 until month 6 among subjects with high titers compared to placebo, but not in subjects with medium and low titers. At month 12, the significant difference in continuous abstinence between participants on placebo and those with high antibody response was maintained.

The finding that significant efficacy is only seen for the high responder group fits with the mechanistic explanation that a sufficient amount of anti-nicotine antibodies is required to sequester the nicotine in the event of a slip. Although based on a subgroup analysis, this suggests a proof of the concept that vaccination against nicotine can increase abstinence rates. High antibody titers were the only factor related to sustained smoking cessation. The separation of abstinence rates already at month 2 between subjects with high titers and those with either low titers or on placebo suggests that not only high titers, but also an early rise of titers seems to be crucial for success. The hypothesis generated from this trial will have to be confirmed in an ITT population in a prospective confirmatory trial.

The vaccine was safe and generally well tolerated. The most prevalent local adverse event was pain at the injection site and the most frequent systemic adverse event was transient flu-like symptoms. While side effects were common, they were self-limited. The clinical trials assessing a VLP-based HPV vaccine also reported mild injection sites reactions [20]. The fact that flu-like symptoms were present in 69% of the subjects on active treatment, as compared to 12.5% of those on placebo, might suggest that the study was essentially unblinded for many subjects. However, there was no difference in the incidence of flu-like symptoms in the 3 groups with low, medium and high antibody titers. The fact that significant abstinence was only achieved in the group with high antibody titers, while medium and low responders had similar abstinence rates as placebo, clearly demonstrates that flu-like symptoms did not influence abstinence rates. Self-reported cigarette consumption and carbon monoxide measurement showed that vaccinated smokers who did not achieve abstinence did not increase smoking to compensate for the potentially lower nicotine amounts reaching the brain in the presence of anti-nicotine antibodies.

Our study results have several limitations. First, whereas the percentage of smoking abstinence (as defined by self-reported smoking status and CO levels smaller than 10 ppm at the monthly visits) in the active group (30.1%) was similar to the one anticipated and used for the sample calculation (30%), the percentage of smoking abstinence in the placebo group (26.1%) was unexpectedly high. The abstinence rate for placebo is similar to that usually found in phase III trials for active treatment using NRT and even higher than the one showed for the bupropion group (20.2%) of the trial comparing efficacy between varenicline, bupropion and placebo [21]. This might be due to differences in the population. Smokers who volunteered for our study had placed very high expectations in the study per se, as illustrated by the very high baseline mean of motivation to stop smoking (8.5 on a visual analog scale from 0 to 10 for subjects in both groups) and confidence in succeeding (7.8 on a visual analog scale from 0 to 10 in subjects randomized to vaccine and 7.7 in those to placebo). In addition, the study participants might have been positively influenced by the quality of the smoking cessation counseling provided by the trained and motivated health care professionals involved in subject recruitment and those involved in the study accomplishment and follow-up [16]. Second, 57 subjects were excluded for the per-protocol analysis based on AUC. However, these excluded subjects represented a minority (19%) and the baseline characteristics of included and excluded subjects were similar, suggesting a low risk of selection bias. Third, we excluded nicotine replacement therapy users because vaccine and nicotine replacement therapy products could be expected to neutralize each other, whereas in smokers on placebo nicotine replacement therapy should exert its positive effect. The main finding of the study was nevertheless not affected when the nicotine replacement therapy users were included, namely that the individuals generating a high antibody response achieved a higher abstinence rate compared to the placebo group.

Immunotherapy has the potential to open a new avenue to the treatment of nicotine addiction [22], [23]. Safety and immunogenicity of a second vaccine, NicVax, has recently been reported in a small non-cessation study involving 14–23 subjects per group [23]. Despite the non-cessation design of the study, if a participant expressed the desire to quit, a brief counseling and a treatment manual was provided. A significantly higher 30-day abstinence, which might have been at any time during the 9-month study, was reported for the highest dose group compared to the placebo group. However, it was not reported whether the proportion of subjects who expressed the desire to quit and made a quit attempt was similar between groups [23].

The antibodies induced by Nicotine-Qβ have high specificity for nicotine and do not cross-react with acetylcholine, the endogenous ligand for nicotinic receptors [9]. In the intention-to-treat analysis the differences in continuous abstinence were non-significant, likely because at the given dose only one third of the subjects achieved sufficient antibody levels. Sufficient binding of nicotine by antibodies is required to reduce the amount of nicotine entering the brain to sub-pharmacological levels. This appears to be critical to block the reinforcing effect of nicotine and thereby to the success of the vaccine. Meanwhile it has been possible to significantly increase anti-nicotine antibody levels and decrease the incidence of flu-like symptoms by reformulating the vaccine (P. Müller, unpublished results). Although a nicotine vaccine is not expected to address all aspects of tobacco dependence [24], our results indicate that antibodies sequestering nicotine in serum might help smokers quit.

## Supporting Information

Protocol S1 Protocol S1.
(0.13 MB PDF)

Checklist S1 CONSORT Checklist.
(0.08 MB DOC)
