# Supplementary material for: A Vaccine against Nicotine for Smoking Cessation: A Randomized Controlled Trial
Source: PLoS One. 2008 Jun 25;3(6):e2547. doi: 10.1371/journal.pone.0002547 (PMC2432028; doi:10.1371/journal.pone.0002547)
Supplement: Protocol S1 — (0.13 MB PDF) [file pone.0002547.s001.pdf]

~~Confidential~~

Clinical Trial Protocol Synopsis

Working document including final **Study Protocol** of 27-Jul-2003, **Amendment I** of 02-Oct-2003, **Amendment II** of 04-Dec-2003 and **Amendment III** of 18-May-2004 and **Amendment IV** of 09-Jun-2004

Evaluation of Efficacy and Safety of Nicotine-Qbeta (NicQb) Vaccine versus Placebo in Healthy Smokers

Sponsor:

Cytos Biotechnology AG

Wagistrasse 25

CH-8952 Schlieren-Zürich

Trial number:	**CYT002-NicQb 02**
Product:	CYT002-NicQb
Development phase	Phase II
Version number:	**final**
Release Date:	**09-Jun-2004**
Replaces version of:	26-May-2004 (draft 1)
Created by:	Joerg Willers, PhD

~~*This information is **strictly confidential**. Clinical investigators solely in connection with a clinical investigation shall only use information contained herein. Information contained herein cannot be disclosed, submitted for publication or used for any purpose other than that mentioned above without the sponsor's prior written authorization. Such information is the property of Cytos Biotechnology AG and should not be photocopied or distributed to other persons without the Sponsor's prior written authorization. Additional copies are to be requested from Cytos Biotechnology AG.*~~

Ethics, Good Clinical Practice and Confidentiality Statement

This study will be conducted in accordance with ICH GCP Guidelines (Directive CPMP/ICH/135/95) and the Declaration of Helsinki (1964) and subsequent revisions.

The Study Protocol and any Amendments, the Investigator's Brochure, the Study Subject Information and Informed Consent Forms as well as other relevant or requested materials such as advertisements must be reviewed by an Independent Ethics Committee or Institutional Review Board.

Informed consent must be obtained from all study subjects before screening. Study subjects must be provided the written Study Subject Information which will then be discussed with the Investigator or his/her deputy. After resolution of all remaining questions the subjects will consent to participate by signing the Informed Consent Form.

Confidentiality of all information received or developed in connection with this protocol must be maintained by the investigators as well as all other personnel involved in the trial who are employed by them.

By signing this protocol, the persons responsible for the conduct of this study confirm that they have read this and agree to conduct the trial as outlined in the protocol.

Study personnel and administrative structure

Investigators:

Center Lausanne<br>(Study Chairman)	PD Dr. Jacques Cornuz<br>Dept of Internal Medicine<br>Institute of Social and Preventive Medicine<br>University Hospital Lausanne<br>Rue du Bugnon 46<br>CH-1011 Lausanne<br>Phone: ++41-(0)21-314-05 06<br>FAX: ++41-(0)21-314-08 71<br>e-mail: Jacques.Cornuz@chuv.hospvd.ch
Center St. Gallen	Prof. Dr. med. Thomas Cerny<br>Head of Oncology / Haematology<br>Kantonsspital St. Gallen<br>Rorschacherstrasse<br>CH-9007 St. Gallen<br>Phone: ++41-(0)71-494-10 62<br>FAX: ++41-(0)71-494-28 78<br>e-mail: thomas.cerny@kssg.ch
	Dr. Felix Jungi<br>Kirchhalde 7<br>CH-9303 Wittenbach<br>Phone/FAX: ++41-(0)71-47 94<br>e-mail: wf.jungi@bluewin.ch

Center Zürich

Dr. Karl Klingler
Lungenzentrum Hirslandenklinik Zürich
Witellikerstrasse 36
CH-8029 Zürich
Phone: ++41 (0)1-387-30 00
FAX: ++41 (0)1-387-22 55
e-mail: klingler@lungenzentrum.ch

The PI at each site will nominate a Co-Investigator who will assist supervising the study. In addition, each site will have at least one medically qualified person (sub-investigator, study physician) and a study nurse or technician fully dedicated to the study and in charge of the organization, clinical conduct and documentation.

Sponsor: Cytos Biotechnology AG

Head of Clinical Development

Philipp Müller, MD
Wagistrasse 25
CH-8952 Zürich-Schlieren
Phone: ++41-(0)1-733-47 12
FAX: ++41-(0)1-733-47 40
e-mail: philipp.mueller@cytos.com

Clinical Trial Leader

Joerg Willers, PhD
Cytos Biotechnology AG
Wagistrasse 25
CH-8952 Zurich-Schlieren
Phone: ++41-(0)1-733-47 50
FAX: ++41-(0)1-733-46 59
e-mail: joerg.willers@cytos.com

Study Monitoring and Data Management:

MDS Pharma Services
6 avenue de la Cristallerie
F-92316 Sèvres Cedex
Phone: ++33-(0)1-46 90 25 30
FAX: ++33-(0)1-46 90 25 25
e-mail: dominique.gerbet@mdsps.com

**Statistician:**	PD MER Guy van Melle, PhD<br>Institute of Social and Preventive Medicine<br>Department of Statistics<br>Rue du Bugnon 17<br>CH-1005 Lausanne<br>Phone: ++41-(0)21-314-72 65<br>FAX: ++41-(0)21-314-73 73<br>e-mail: guy.van-melle@inst.hospvd.ch
**Study Sites:**	Kantonsspital St. Gallen<br>Rorschacherstrasse<br>CH-9007 St. Gallen<br><br>University Hospital Lausanne (CHUV)<br>Rue du Bugnon 46<br>CH-1011 Lausanne<br><br>Lungenzentrum Hirslandenklinik Zürich<br>Witellikerstrasse 36<br>CH-8029 Zürich

Protocol Synopsis

Title:

Evaluation of Efficacy and Safety of Nicotine-Qbeta (NicQb) Vaccine versus Placebo in Healthy Smokers

Development Phase:

Phase II

Objectives:

The objectives of this exploratory Phase II trial are

- To assess **clinical efficacy** of NicQb in smokers willing to quit
  - by determination of 3 months *continuous abstinence rate* at week 24, based on smoking diaries and urinary cotinine;
  - by determination of *point-prevalence of abstinence* during the past 24 hours at monthly intervals, from the target quitting date up to week 24, based on self-reported smoking status and carbon monoxide in exhaled air;
  - by recording *changes in smoking habit*, at monthly intervals from the target quitting date up to week 24, based on diaries.
- To evaluate **safety and tolerability** (local and systemic) of NicQb in healthy smokers
  - by frequent monitoring of *clinical signs and symptoms* up to week 24
  - by assessing smoker's *psychological state* (craving, withdrawal symptoms, mood) through appropriate questionnaires
- To determine **immunogenicity** of NicQb
  - by determination of titers and titer profiles of *specific anti-nicotine antibodies* (Ab) in serum from pre-dose in monthly intervals up to weeks 24, and during an open follow-up phase up to 12 months

Study Design:

This will be a randomized Phase II efficacy and safety study with one dose of NicQb versus placebo, conducted according to a parallel-group, multi-center study design. At the first center starting the trial, the inclusion of subjects will be staggered such that the first 20 subjects must have completed visit 2 (7 days after the first injection) and tolerability shown to be good; then the additional subjects may be included as they are available and eligible.

Selected Dose:

The dose of 100ug NicQb+Alum has been selected based on the results from the Phase I study. The highest dose used in Phase I was 100µg of the vaccine, tested with and without Alum. All doses have been proven to be well tolerated. Therefore, the immunogenicity of the different doses served as a parameter for dose selection. Since the titer of specific anti-nicotine antibodies is expected to be closely related to the efficacy of the vaccine, a dose of 100µg+Alum was chosen based on its early onset and maximal immune response observed in the Phase I study.

Study Subjects:

300 otherwise healthy smokers (defined as adults between 18 and 70 years – male and female – who have been smoking for more than 3 years ≥ 10 and ≤ 40 cigarettes/day), with a Fageström Index of ≥ 5 at screening, and motivated to quit smoking will be enrolled into the study after written informed consent has been obtained Woman of child-bearing potential must use an effective means of contraception up to 12 months after the last dose of the vaccine.

Exclusion criteria: cardiovascular, renal, pulmonary, endocrine, or neurological disorders, ulcers, dermatologic disorders, autoimmune diseases or severe allergies; an active infectious disease (HBV, HCV); a current diagnosis or a history of relevant depressive episodes or of panic attacks, psychosis, bipolar or eating disorders; use of other smoking-cessation treatments, - like bupropion or nicotine-replacement therapy - within 6 months before study enrolment; pregnancy or lactation; abuse of alcohol or other recreational drugs; use of a psychoactive drug (excluding sleeping pills) within one month before enrolment, and regular use of any non-cigarette tobacco product. Significant clinical laboratory findings (blood chemistry, hematology, urinalysis, ECG).

Study Procedures:

Eligible smokers will be randomized to receive either an active NicQb dose of 100µg+Alum (n=200) or indistinguishable placebo injections (n=100), followed after 4, 8, 12 and 16 weeks by respective boost injections. Efficacy will be recorded at the end of the study after 6 months for continuous abstinence, and monthly during the study after the prime dose for point prevalence abstinence and changes in smoking habits.

Target quitting date (TQD) will be at Visit 4, on the day of the second injection (i.e. 4 weeks after the first injection). Starting one week before TQD participants will attend brief individual counseling sessions for smoking cessation. Smoking habits will be recorded based on diaries. Questionnaires to assess craving and withdrawal symptoms including mood (signs of depression) will be filled out. Blood samples will be taken for routine clinical laboratory, for Ab titer determinations, and a urine sample will be obtained for measuring cotinine levels.

There will be a screening visit, 4 treatment application visits for the vaccine or placebo injections, each followed by a safety check-up one week later. Additionally, there will be 6 visits during the double-blind phase of the trial for the assessment of efficacy parameters and/or brief counseling.

After completion of the double-blind 6-months trial phase the study subjects are requested to return for additional follow-up visits at 9 and 12 months after the prime dose for additional blood sampling to monitor Ab titers and to obtain information on current smoking habits. Those visit results will not enter into the primary efficacy evaluation but serve exploratory purposes regarding long-term efficacy and pharmacodynamics.

Route of Administration:

The study drug will be injected by the intramuscular (i.m.) route. The injection volume will be 2ml including the adjuvant which is added to Qb prior to injection. The volume and mode of administration will be optimized for further clinical development. The injection site for the prime dose will be the right deltoid muscle and only when necessary changed to the gluteal muscle. Alternating sides will be used for the boost injections.

Active Pharmaceutical Ingredient (API):

- Nicotine-Qbeta (nicotine coupled to Virus-like Particle (VLP) as a carrier)
- Quality: GMP
- Manufacturer: Cytos Biotechnology AG

Drug Product (DP):

- Concentration: 75ug of API per 1ml of PBS buffer
- Quality: GMP
- Volume per drug vial: 1.5ml

Adjuvant:

Acetate-buffered Alhydrogel (Alum)

Comparator:

Placebo, PBS buffer

Study Endpoints:

- <u>Clinical efficacy:</u>
  - *Continuous abstinence* is assessed at 6 months (week 24) after the prime dose and is defined as abstinence during a 3 months period starting at Visit 13 (week 12) till Visit 18 (week 24). Evaluation is based on self-reported number of cigarettes smoked during that time interval based on diaries, and supported by urinary cotinine and exhaled carbon monoxide determined on the occasion of the respective visits.
  - *Point-prevalence of abstinence* is determined monthly up to 6 months after the prime dose, by reported non-smoking status for 24 hours on the day of assessment, and confirmed by a carbon monoxide concentration in expired air of 10ppm or less
  - *Changes in smoking habits* are recorded monthly up to 6 months after the prime dose, by self-reported number of cigarettes smoked (diary) and possible

switches in brand; these data will be compared to a baseline recording which starts two weeks before the first injection.

- Safety and tolerability is assessed through systematic collection of vital signs, standard clinical laboratory exams (hematology, blood chemistry, urinalysis and ECG) and all reported symptoms in all subjects. Craving and withdrawal symptoms including mood will be assessed through appropriate questionnaires.
- Immunogenicity will be assessed in all subjects by measuring anti-nicotine Ab titers in serum from pre-dose at monthly intervals up to 6 months after the prime dose, and will be followed up in all subjects during an open follow-up period up to 12 months after the prime dose.

Study Duration:

- enrolment phase: 4 to 6 months, planned start October 2003
- double-blind treatment phase: 6 months
- open-label optional follow-up phase: 6 months
- evaluation and reporting period: 6 months

Study Sites:

I) Kantonsspital St. Gallen
II) University Hospital Lausanne
III) Lungenzentrum Hirslandenklinik, Zürich
IV) Klinik AndreasKlinik, Cham Zug

Statistical Considerations:

Studies with nicotine replacement therapy and bupropione have shown statistically significant separation between active and placebo at 6 and 12 months follow-up in the range of 10% -20%.

Since this Phase II study should lead to a clinical proof of concept (efficacy in smoking cessation) it is powered to detect at least a 15% difference from placebo in 3-months continuous abstinence rate assessed at 6 months after the prime vaccination, with a statistical significance of $p<0.05$ at a power of 90%. With a 2:1 ratio of subjects on active drug : placebo, the trial will require 300 subjects to participate. All subjects that have received the prime dose will be eligible for the intent-to-treat analysis for efficacy.

Considering the exploratory nature of this Phase II study additional evaluations may be considered driven by the results, since very little is known about the inter-subject variability of responses to the vaccine and even less about the clinical outcome and its correlation with Ab titers.

Total Blood Volume per Subject:

200ml within one year (80ml during the 1st month, 100ml during 2nd to 6th month, 20ml during 7th to 12th month.

Trial Flow Chart

TQD ⇓ (at V 4)

	Pre- Study	Visit 1 day 0 wk 0	V 2 d 7 wk 1	V 3 d 21 wk 3	V 4 d 28 wk 4	V 5 d 35 wk 5	V 6 d 42 wk 6	V 7 d 49 wk 7	V 8 d 56 wk 8	V 9 d 63 wk 9	V 10 d 70 wk 10	V 11 d 77 wk 11	V 12 d 84 wk 12	V 13 d 91 wk 13	V 14 d 112 w k	V 15 d 119 wk 17	V 16 d 140 wk 20	V 17 d 168 wk 24	V 18 d 252 wk 36	V 19 d 364 wk 52
**Injection of Study Drug**		**X**			**X**				**X**				**X**		**X**				Follow-up	
Continuous abstin. end-point ├──┤<br>Point prevalence end-points •		•			•				•				├ •<br>(V 12 – V 17)	─	─ •	─	─ •	┤ •		
Smoking Cessation Counseling				X	X	X	X	X	X	X	X	X	X	X	X					
Smoking Diary collection		X	X	X	X	X	X	X	X	X	X	X	X	X	X		X	X		
Carbon Monoxide		X			X				X				X		X		X	X	X	X
Cotinine (urine)		X			X				X				X		X		X	X	X	X
Craving, Withdrawal Sympt. and Mood		X			X				X				X		X		X	X		
Informed Consent	X																			
Fageström (FTND)	X																	X		
Smoking History	X																			
Abuse of Drugs and Alcohol Screen	X																			
HBV, HCV	X																			
Baseline Ig Assessm. (IgA, IgG, IgM)	X																			
Complete Med/Surg History	X																			
* Interim Medical History		X			X				X				X		X			X		
Complete Physical Exam and ECG	X																	X		
Interim Physical Exam		X			X				X				X		X					
Injection Site Examination			X		X	X			X	X			X	X	X	X				
Vital Signs	X	X			X				X				X		X			X		
Haematology	X	X			X				X				X		X			X		
Blood Chemistry and Urinalysis	X																	X		
Serum for anti-nicotine Ab assessment		X			X				X				X		X		X	X	X	X
** Beta-hCG Pregnancy Test	X	X			X				X				X		X			X		

* Additionally, the subject is asked to report by telephone about any side effects occurring between the visits
** Only females of childbearing potential
