# Supplementary material for: A Vaccine against Nicotine for Smoking Cessation: A Randomized Controlled Trial
Source: PLoS One. 2008 Jun 25;3(6):e2547. doi: 10.1371/journal.pone.0002547 (PMC2432028; doi:10.1371/journal.pone.0002547)
Supplement: Checklist S1 — CONSORT Checklist. (0.08 MB DOC) [file pone.0002547.s002.doc]

CONSORT **Checklist**

# a vaccine against nicotine for smoking cessation

**a phase II randomised controlled trial**

J Cornuz MD, S Zwahlen MD, W F Jungi MD, J Osterwalder MD, K Klingler MD, G van Melle PhD, Y Bangala, I Guessous MD, P Müller PhD, J Willers PhD, P Maurer PhD, M F Bachmann PhD, T Cerny MD.

CONSORT **Checklist**

| **PAPER SECTION And topic** | Item | Description and Comments related to the study | **Reported on** Page # |
| --- | --- | --- | --- |

| ***TITLE & ABSTRACT*** | 1 | How participants were allocated to interventions  (*e.g*., "random allocation", "randomised", or "randomly assigned"). | | | |
| --- | --- | --- | --- | --- | --- |
|  |  | **We report the term *randomised controlled trial* in both the title and the abstract.** | Title, abstract | | |
| ***INTRODUCTION* Background** | 2 | Scientific background and explanation of rationale. | | | |
|  |  | **-The first part of the introduction focuses on the problem  of smoking habits and nicotine dependence.**  **-We then describe the scientific rationale of immunization against nicotine.**  **-We present the previous animal and human studies results using immunization and suggesting that this intervention might work.**  **-To highlight that the intervention (i.e. vaccine injection) would not exposed participants to unnecessarily risks (Helsinki Declaration), we present the results of a phase I study including 32 participants and showing that the vaccine was well tolerated.**  **-Finally, we expose in the introduction part that the goals of this study was to assess the safety, tolerability, immunogenicity, and efficacy of the vaccine against  nicotine** | Introduction:  1^st^ paragraph  Introduction:  2^nd^ paragraph  Introduction:  3^rd^ paragraph  Introduction:  4^th^ paragraph  Introduction:  5^th^ paragraph | | |
| ***METHODS* Participants** | 3 | Eligibility criteria for participants and the settings and locations where the data were collected. | | | |
|  |  | **-The first part of the method section describes the method of recruitment, which consisted of *public advertisements on billboards posted in three clinical study centres*.-The second part of this section thoroughly describes the selection criteria and lists all the exclusion criteria used.** | Methods:  1^st^ paragraph  Methods:  2^nd^ paragraph | | |
| **Interventions** | 4 | Precise details of the interventions intend for each group and how and when they were actually administered. |  | | |
|  |  | **-Vaccine characteristics and its mode of administration are explained in the methods section.**  **In addition to vaccine and placebo injection procedures,  we also describe the other interventions that constitute the usual care given to both control and intervention group. Indeed, safety and tolerability assessment through systematic collection of vital signs, standard clinical laboratory, injection site examinations, specific safety check-up performed, and subjects’ diary for  self-assessment of local reactions are all described in this section.**  **-Details of the timing and duration of interventions are reported in this section.** | Methods:  Randomization and interventions  Methods:  Randomization and interventions | | |
| **Objectives** | 5 | Specific objectives and hypotheses. | | | |
|  |  | **-The hypotheses and objectives are specifically named and separately described in the manuscript.** | Methods:  Design overview | | |
| **Outcomes** | 6 | Clearly defined primary and secondary outcome measures and,  when applicable, any methods used to enhance the quality of  measurements (*e.g.*, multiple observations, training of assessors). | | | |
|  |  | **-A specific section within the method paragraph describes the outcomes. We distinctly present the primary outcome (*abstinence from smoking*) and the secondary outcomes (i*mmunogenicity,* *safety and tolerability*).**  **- biochemical confirmation of self-reported abstinence, antibodies determination procedure, timing of determination are presented.** | Methods:  Outcomes and follow-up  Methods:  Outcomes and follow-up | | |
| **Sample size** | 7 | How sample size was determined and, when applicable,  explanation of any interim analyses and stopping rules. | | | |
|  |  | **-The statistical analyses section included information’s concerning how sample size was determined.** | Methods:  Statistical analyses | | |
| **Randomisation -- Sequence generation** | 8 | Method used to generate the random allocation sequence,  including details of any restrictions (*e.g*., blocking, stratification) – See below - point 10 | | | |
| **Randomisation -- Allocation concealment** | 9 | Method used to implement the random allocation sequence  (*e.g*., numbered containers or central telephone), clarifying  whether the sequence was concealed until interventions were  assigned. See below – point 10 | | | |
| **Randomisation -- Implementation** | 10 | Who generated the allocation sequence, who enrolled participants, and who assigned participants to their groups? | |  | |
|  |  | **-Random allocations, sequence generation and implementation (block, local pharmacist, software) are described in the method section** | Methods:  Randomization and interventions | | |
| **Blinding (masking)** | 11 | Whether or not participants, those administering the interventions, and those assessing the outcomes were blinded to group assignment. When relevant, how the success of blinding was evaluated. |  | | |
|  |  | **-Study personnel, including statisticians and participants blinding are explicitly reported in the manuscript** | Methods:  Randomization and interventions | | |
| **Statistical methods** | 12 | Statistical methods used to compare groups for primary  outcome(s); Methods for additional analyses, such as  subgroup analyses and adjusted analyses. | |  | |
|  |  | **-Comparisons of proportions, continuous variables differences are described, as well as the rationale to use logistic regression** | Methods:  Statistical analyses | | |
| **RESULTS**  **Participant flow** | 13 | Flow of participants through each stage (a diagram is strongly recommended). Specifically, for each group report the numbers of participants randomly assigned, receiving intended treatment, completing the study protocol, and analysed for the primary  outcome. Describe protocol deviations from study as planned, together with reasons. | |  | |
|  |  | **-A diagram of participants flow through each stage is illustrated on figure 1**  **-We explain the exact reason to exclude participants. We summarized this information in the flow chart diagram** | Figure 1  Figure 1 | | |
| **Recruitment** | 14 | Dates defining the periods of recruitment and follow-up. | |  | |
|  |  | -**We precise the duration of the enrolment process.** | Results:  Participants | | |
| **Baseline data** | 15 | Baseline demographic and clinical characteristics of each group. | |  | |
|  |  | **-Information is presented in the text and in table 1.** | Results  Table 1 | | |
| **Numbers analysed** | 16 | Number of participants (denominator) in each group included in each analysis and whether the analysis was by "intention-to-treat". State the results in absolute numbers when feasible (*e.g*., 10/20, not 50%). | |  | |
|  |  | **-Number of participants in each group included in each analysis are systematically described in the findings section (e.g. re. immunogenicity and smoking abstinence outcome, 229 and 111 participants taking into consideration, in the intervention and control group respectively; for analysis after excluding nicotine replacement therapy users: 199 and 97 participants taking into consideration; for per protocol analysis, 159 and 80 participants taking into consideration)** | Abstract:  Methodology/  principal findings | | |
| **Outcomes and estimation** | 17 | For each primary and secondary outcome, a summary of results for each group, and the estimated effect size and its precision (*e.g.*, 95% confidence interval). |  |  | |
|  |  | **-We report results of both primary and secondary outcomes. These results are reported in the text, tables and figures. We use confidence intervals to judge the precision level. In addition to confidence intervals we report p-value. For adverse events we report odds ratio and confidence intervals** | Results  Tables 2, 3, 4, 5  Figures 2,3, 4, 5 | |  |
| **Ancillary analyses** | 18 | Address multiplicity by reporting any other analyses performed, including subgroup analyses and adjusted analyses, indicating those pre-specified and those exploratory. | |  | |
|  |  | **-As mentioned in the protocol, analysis of kinetics of the immune responses was planned, as well as the relation between immune response and smoking abstinence. We chose unbalanced group sizes with a ratio of 2:1 for active vs. placebo treatment to permit sub-group analyses** | Methods:  Smoking  behaviour in the per protocol analysis | |  |
| **Adverse events** | 19 | All important adverse events or side effects in each  intervention group. | | |  |
|  |  | **-We estimate the frequency of adverse events separately for each group. We report them in the text and tables 3 and 4. We also provide operational definitions for the measures of the severity of adverse events.** | Tables 3, 4 |  | |

| **DISCUSSION Interpretation** | 20 | Interpretation of the results, taking into account study hypotheses,  sources of potential bias or imprecision and the dangers associated with multiplicity of analyses and outcomes. | | |
| --- | --- | --- | --- | --- |
|  |  | **-In the discussion section, we follow a structured organization. We first present a synopsis of the key findings, and then we discuss the four main limitations of the study as well as source of potential bias. We finally describe the potential implication of nicotine vaccine to help smokers quit.** | Discussion |  |
| **Generalisability** | 21 | Generalisability (external validity) of the trial findings. | |  |
|  |  | **-Because the current study is a phase II study, we do not address generalisability of the trial findings.** |  |  |
| **Overall evidence** | 22 | General interpretation of the results in the context of current evidence. | |  |
|  |  | **-We describe the potential implication of nicotine vaccine to help smokers quit.** | Discussion:  Last paragraph |  |
